# Biomechanical explanation of W-plasty effectiveness using a finite element method approach

**DOI:** 10.1038/s41598-023-45400-z

**Published:** 2023-10-23

**Authors:** Marios Papadakis, Georgios Manios, Georgios Zacharopoulos, Dimitra Koumaki, Andreas Manios

**Affiliations:** 1 Department of Surgery II, University of Witten-Herdecke, Heusnerstr. 40, 42283 Wuppertal, Germany; 2 Department of Computer Science and Biomedical Informatics, University of Thessaly, Volos, Greece; 3 Department of Surgical Oncology, School of Medicine, University Hospital Heraklion, Heraklion, Greece; 4 Department of Dermatology, School of Medicine, University Hospital Heraklion, Heraklion, Greece

**Keywords:** Biophysics, Medical research

## Abstract

The finite element method has often been used to assist analyzing local flaps in terms of deformation and stress measurements as it takes into account complex skin properties. We, herein, present an isotropic two-dimensional finite element skin model applied to the W-plasty method to demonstrate that the good outcomes of W-plasty should be attributed to the geometry itself, as it generates lower stresses. The skin was modeled as a two-dimensional (2D) planar geometry. The model was created and solved as a plane stress problem. The model was based on simulation of the loading and stitching of W-plasties of various angles. Each central triangular flap was segmented in nine triangular elements. The stitching was modeled with one suture at the top of each triangular flap with the center of the opposite corner. X- and Y-axis stresses and shearing stresses Txy in the elements involved in the broken stitching line, show lower stresses than the elements behind the stitching line. Interestingly, in the triangular flaps, the stresses were clearly lower than those of their neighboring areas. The maximum compressive stresses in the 2D model we used, correspond to the dog ears. We conclude that the effectiveness of W-plasty should be attributed not only to the scar orientation in relation to the relaxed tension skin lines but also to the special design of the triangular flaps used. This finding assists the general understanding of the method and should be taken into account by the clinician during flap designing.

## Introduction

Wound closure is a biomechanical problem^1^. After closure, strains and stresses around the wound will change, leading to hypertrophic scars in areas of excessive tension^1^. Geometric broken-line closure techniques have traditionally been used to revise contracted and hypertrophic scars, scars with poor skin length matching and scars that cross the relaxed skin tension lines in a perpendicular fashion.

The Cuban plastic surgeon Albert Borges was the first to publish a treatise on a W-shaped broken-line closure technique in 1959^2^. However, it is worth noting, that a similar technique had already been described by Ombredanne in 1937 to correct a congenital constrictive band of the lower limb^3^. According to Borges, the Chilean plastic surgeon Paul Covarrubias was the first to have conceived the idea of a zigzag pattern using small triangular cutaneous flaps that follow the direction of the skin tension lines. Covarrubias presented his method at the 7th congress of the Latin American Society of Plastic Surgery in 1954 under the title “Original Technique in the Treatment of Facial Scars”^2^. Borges named the method “W-plasty” after the outline of the incision, which resembled that of various W’s put together. Its main advantage is that it reflects light more poorly than linear scars. Therefore, the indication for W-plasty is a scar on flat facial surfaces, e.g. cheek or between the lower lip and the jaw, while W-plasty is not suitable for scars on major joints, e.g. axilla and elbow^4^. Wang et al. has found W-plasty to be more effective when combined with continuous tension-reduction, i.e. tension offloading devices such as steri-strips^5^.

W-plasty has several advantages over Z-plasty, the greatest being, that, after flap transposition, the arms of Z do not follow the lines of skin tension as well as do the arms of the W^2^. Rather than a running Z-plasty, W-plasty is not applied to lengthen the scar^6^. For these reasons, W-plasty has recently gained increasing attention, and many attempts have been made to improve its design^4–9^. Jáuregui et al. demonstrated W-plasty to be superior to the traditional straight-line closure in terms of scars in patients undergoing paramedian forehead flaps^6^. Goutos et al. report the geometric design of W plastics in various parts of the body. Unfortunately, all published studies failed to provide an adequate explanation for its superiority. The first attempt to mathematically explain the effectiveness of W-plasty was made by James Fleming and Horace Williams^10^, who used simple geometric models. However, these models are proven to be insufficient when used to describe elastic bodies that exhibit stresses and deformations. Moreover, we are still unable to quantitatively predict skin stress and deformation during wound closure in everyday clinical practice^11^.

The finite element method has often been used to assist analyzing local flaps in terms of deformation and stress measurements^1,11–15^. Its main advantage is that it takes into account complex skin properties, such as anisotropy and nonlinearity, which geometric design alone does not^1^. However, to the best of our knowledge it has not been applied to the W-plasty method.

We, herein, present an isotropic two-dimensional finite element (2D FE) skin model applied to the W-plasty method to demonstrate that the good outcomes of W-plasty should be attributed to the method itself, as it generates lower stresses. We used an isotropic model to assess the developed tensions after flap stitching, considering this a plane stress problem. According to our hypothesis, if tension reduction is applied to an isotropic model, a higher reduction is expected when applied to the anisotropic skin. If W-plasty reduced the tension in the triangular flaps, this would mean that the superior W-plasty related results should be attributed not only to more favorable scar orientation but also the geometry of the technique itself.

Our investigation into the effectiveness of W-plasty in wound closure through finite element analysis stems from a pressing need to elucidate the biomechanical underpinnings of this surgical technique. While the finite element method has proven invaluable in analyzing local flaps, particularly in terms of deformation and stress measurements, its application to W-plasty has been conspicuously absent from prior research. This knowledge gap is noteworthy considering the intricate dynamics at play in W-plasty, which involves the strategic arrangement of triangular flaps.

## Methods

### Software

The model was created and solved as a plane stress problem with the non-commercial finite element (FE) program NLFED developed by the senior author. This software consists of two parts: one part prepares the data to be entered into the second part, which solves the problem. The same first part of the program handles the post-processing and provides the results in a user-friendly and understandable way. The initial version of the program was written in 1992 in Visual Basic 1.0, and the final version in Visual Basic 6.0. This program is for research use only and is not available on the internet. The data is prepared through a user-friendly graphical interface, where node displacements and loads on the nodes are inputted and then saved in a special format. Problems involving up to 10,000 elements can be analyzed. The solutions to the problems are also displayed in the same graphical interface.

### Model

**Figure 1:**
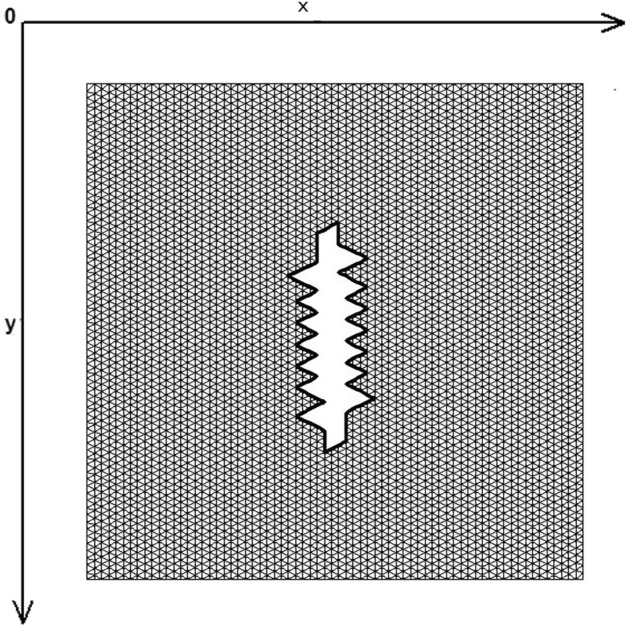
The FE model: incision line and defect after scar removal. The angle of the W-plasty was approximately 55 degrees, while the base angles were about 62.5 degrees.

The skin was modeled as a two-dimensional (2D) planar geometry. The model was based on simulation of the loading and stitching of W-plasties of various angles. The 2D FE model is depicted in Fig. 1 and consisted of 4770 nodes and 9150 triangular elements. The material properties of skin were modeled as nonlinear, elastic and isotropic. Young’s modulus of elasticity E from 7.5 kPa to 15 and Poisson Ratio ν = 0.5. In this case we selected E = 7.5 kPa = 7.5 × 104 dyn/cm^2^ while the thicknes of elastic material was 0.15 cm.

### Boundary conditions

In previous studies of local flaps using the finite element method, no clear method for modeling the stitching was described. We assumed that the sutures were placed simultaneously in the model. While this may seem unrealistic, it’s important to note that the order of the sutures has a significant impact on the resulting tension after stitching. For example, if we use four sutures, we will have 24 stitching permutations, and with five sutures, we will have 120 different permutations, as changing the order of the sutures yields different solutions to our problem. The formula applied to find the number of possible stitching combinations is ‘n factorial (n!)’, where ‘n’ is the number of sutures to be used.

Firstly, we defined which nodes would remain fixed during stitching (boundary conditions). Then, we defined the pairs of nodes that will be stitched. Initially, we arbitrarily chose stitching positions located at some intermediate position between the pair of nodes to be stitched, usually at the midpoint of the distance between them. These initial displacements, together with the fixed nodes, constituted the boundary conditions of the problem and are provided as input data. We then solve the problem with our program.

After the problem was solved, half of the nodes that were ‘stapled’, were removed from the construction, while the other half remained. The elements were redefined with the remaining nodes, and the nodal reactions (forces) developed on the stitched nodes after the solution were used as new loads for the modified structure. We then run the program again, this time using the developed loads on the joined nodes as data. In this second solution, the nodes reached their final positions. If ‘k’ is the number of nodes stapled and ‘n’ is the total number of nodes, the nodes in the modified structure are ‘(n − k)/2’, while the number of elements remained the same.

We also assumed that the sutures were not deformed and that they were placed on the edges of the operative wound.

Institutional Review Board approval was not required in this study as this is a computational study that did not use human subjects.

## Results

**Figure 2:**
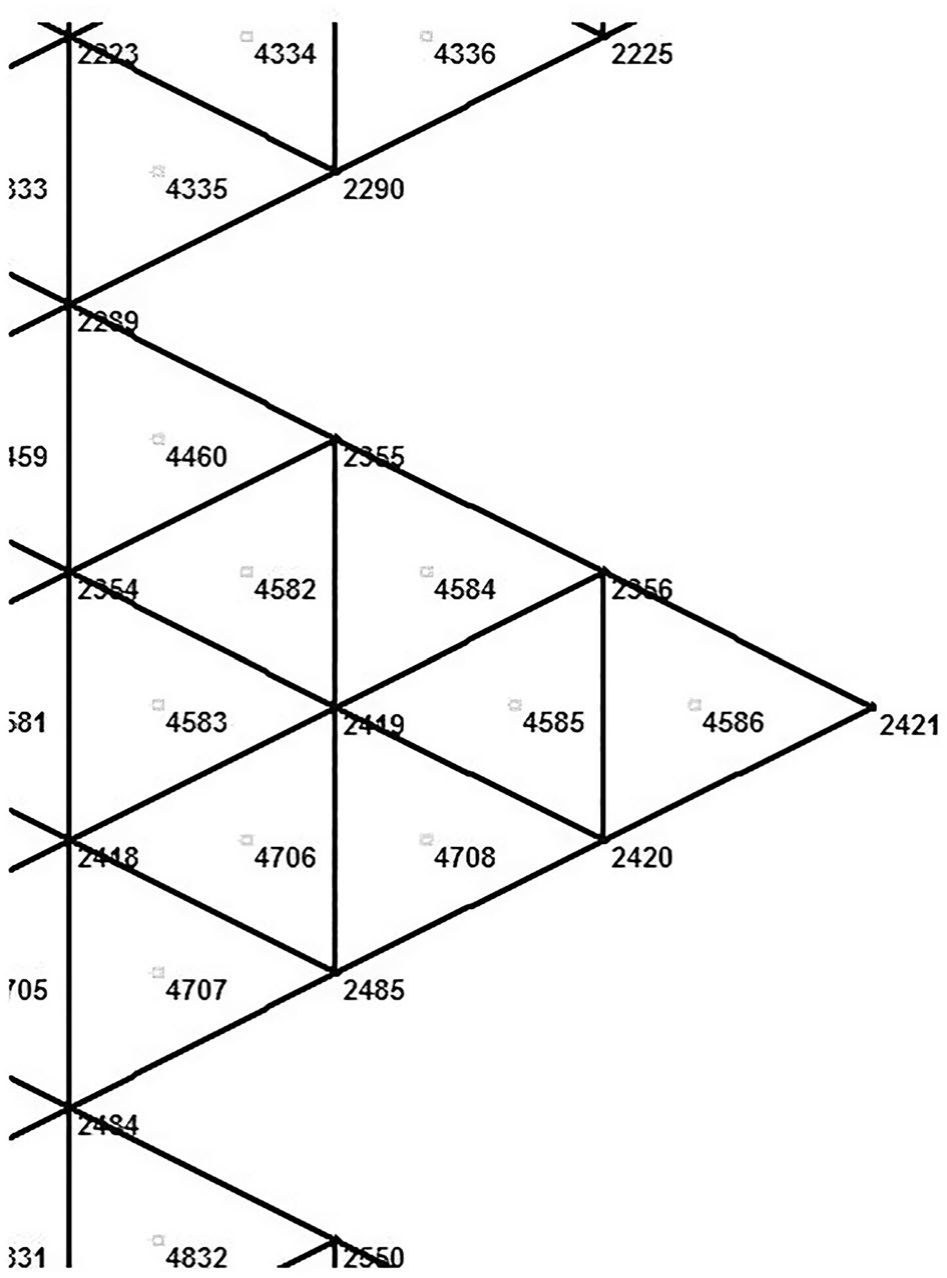
Elements per flap: each triangular flap was segmented in nine triangular elements.

**Figure 3:**
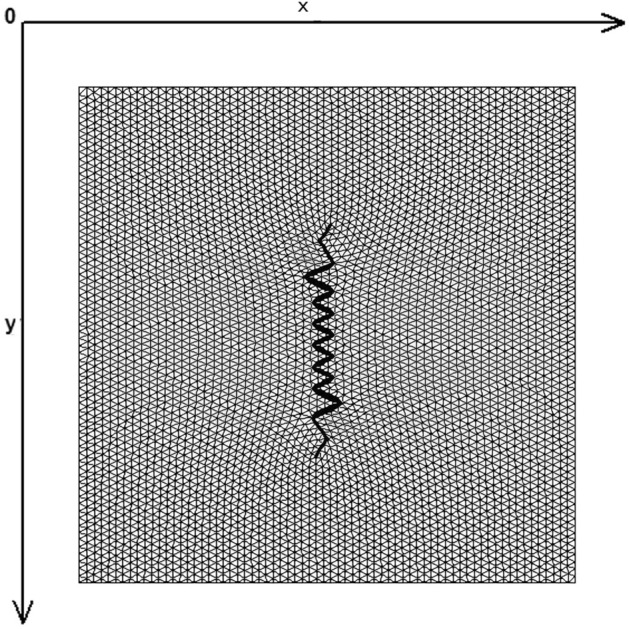
FE model after closure of the defect.

Figure 1 demonstrates the incision line and the deficit created after scar removal. The angle of the W-plasty was approximately 55 degrees while the base angles were about 62.5 degrees, approaching the design proposed by Borges in his classic article^2^, who proposed isosceles triangles each with approximately 6 mm at its base, 6½ mm in height, two 65 degree angles and one 50-degree angle. Each central triangular flap was segmented in nine triangular elements (Fig. 2). Figure 3 shows the elements after stitching. The stitching was modeled with one suture at the top of each triangular flap with the center of the opposite corner, as recommended by Borges^2^.

**Figure 4:**
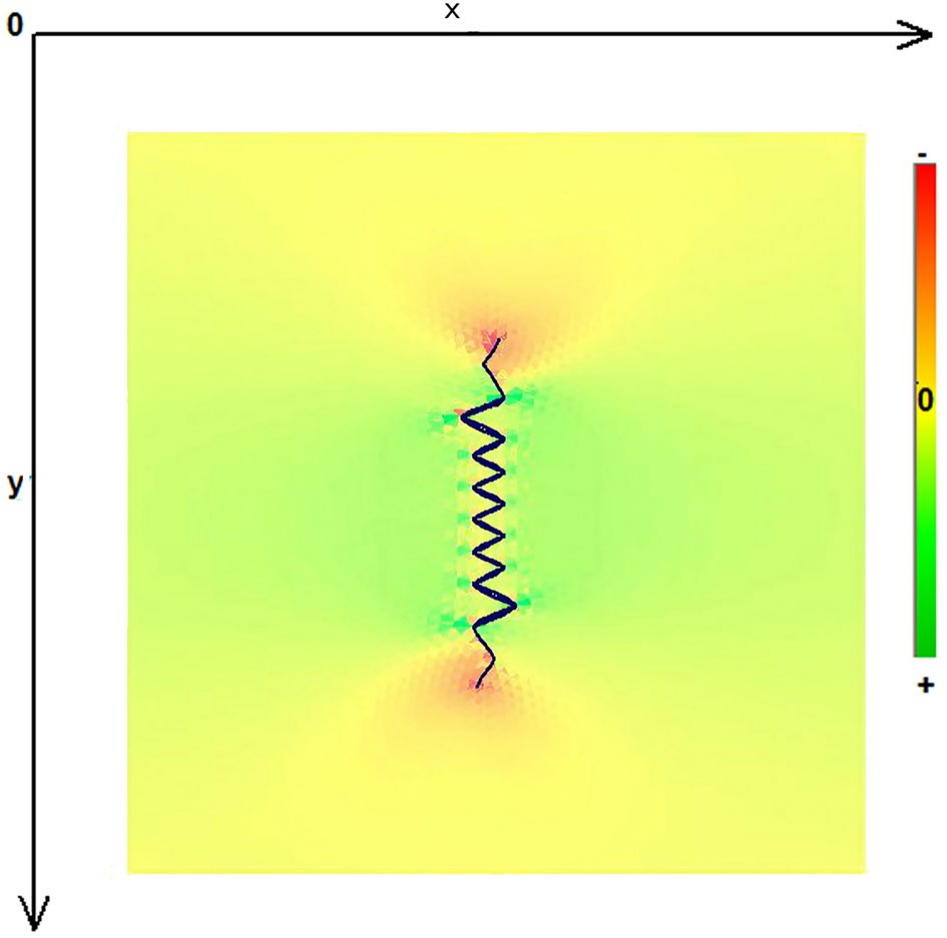
Stress along the x-axis. Compressive stresses are depicted in red, normal stresses are depicted in green and the yellow areas represent low to zero stress areas.

**Figure 5:**
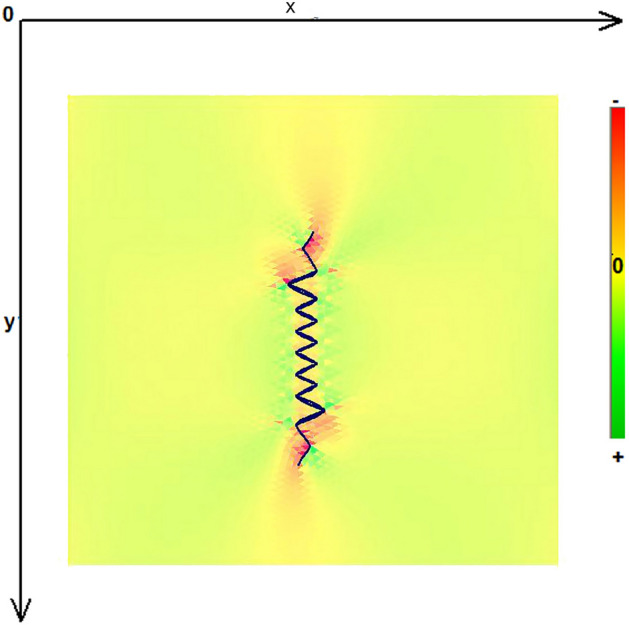
Stress along the y-axis. Compressive stresses are depicted in red, normal stresses are depicted in green and the yellow areas represent low to zero stress areas.

**Figure 6:**
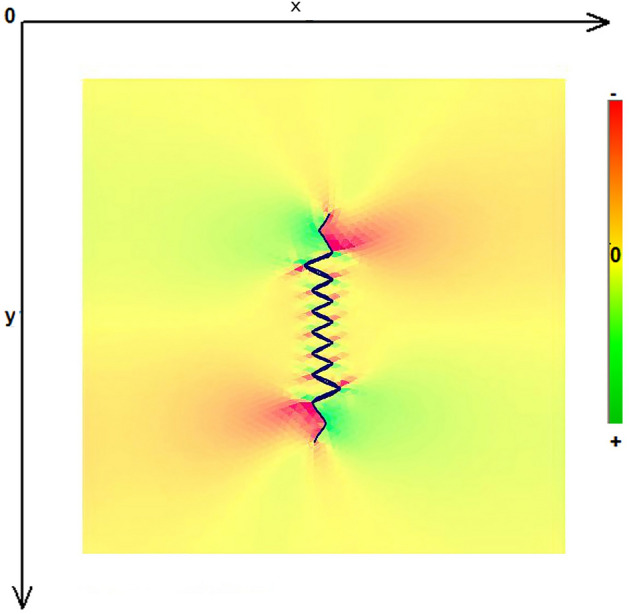
Shearing Txy stress. Compressive stresses are depicted in red, normal stresses are depicted in green and the yellow areas represent low to zero stress areas.

**Figure 7:**
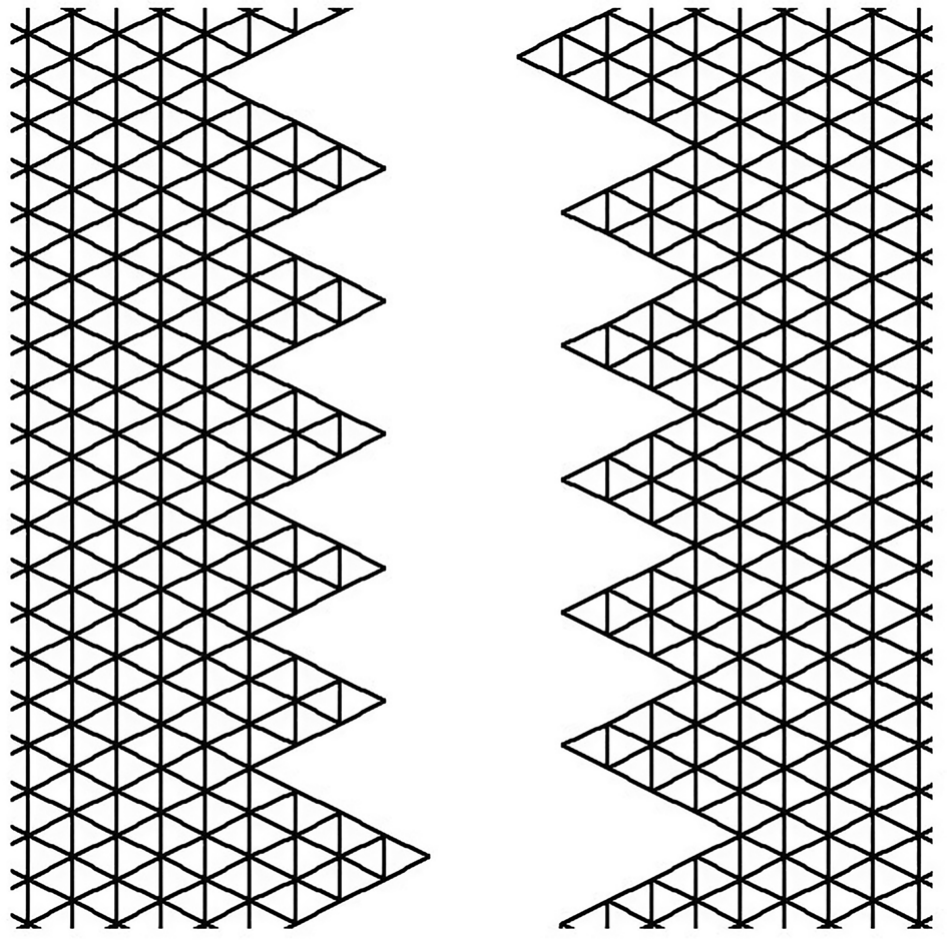
The FE model: zoom at the center before closure.

**Figure 8:**
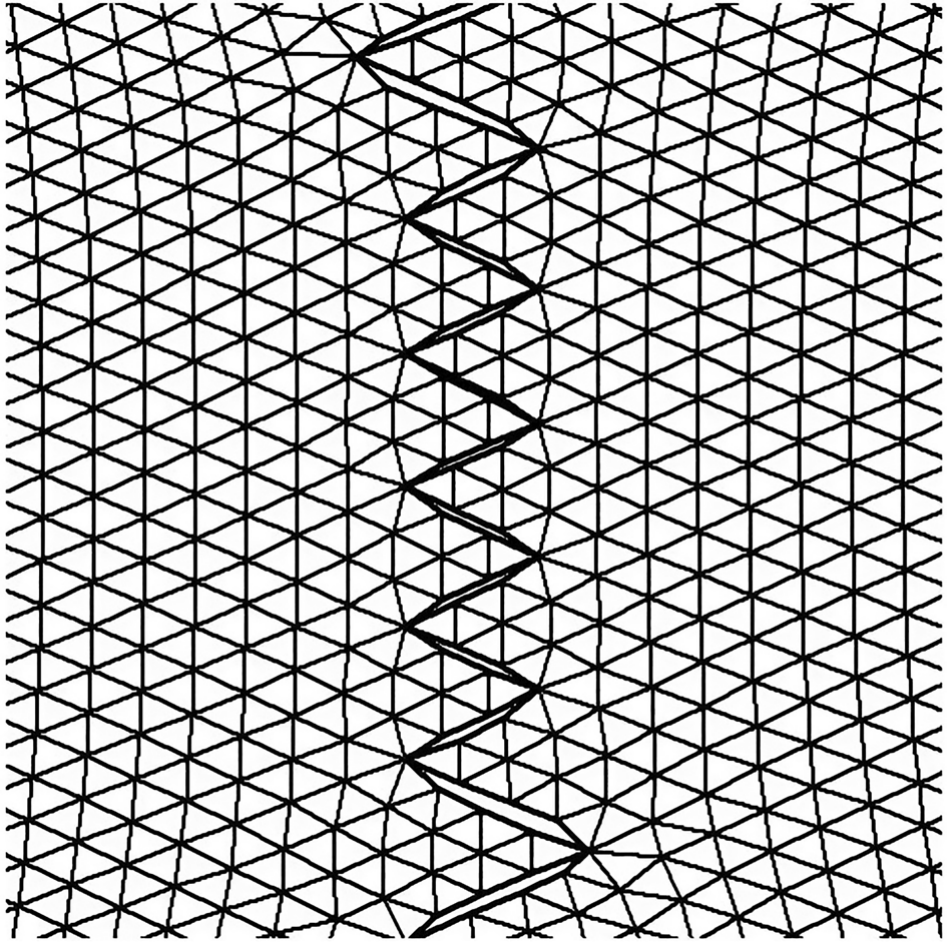
The FE model: zoom at the center after closure.

**Figure 9:**
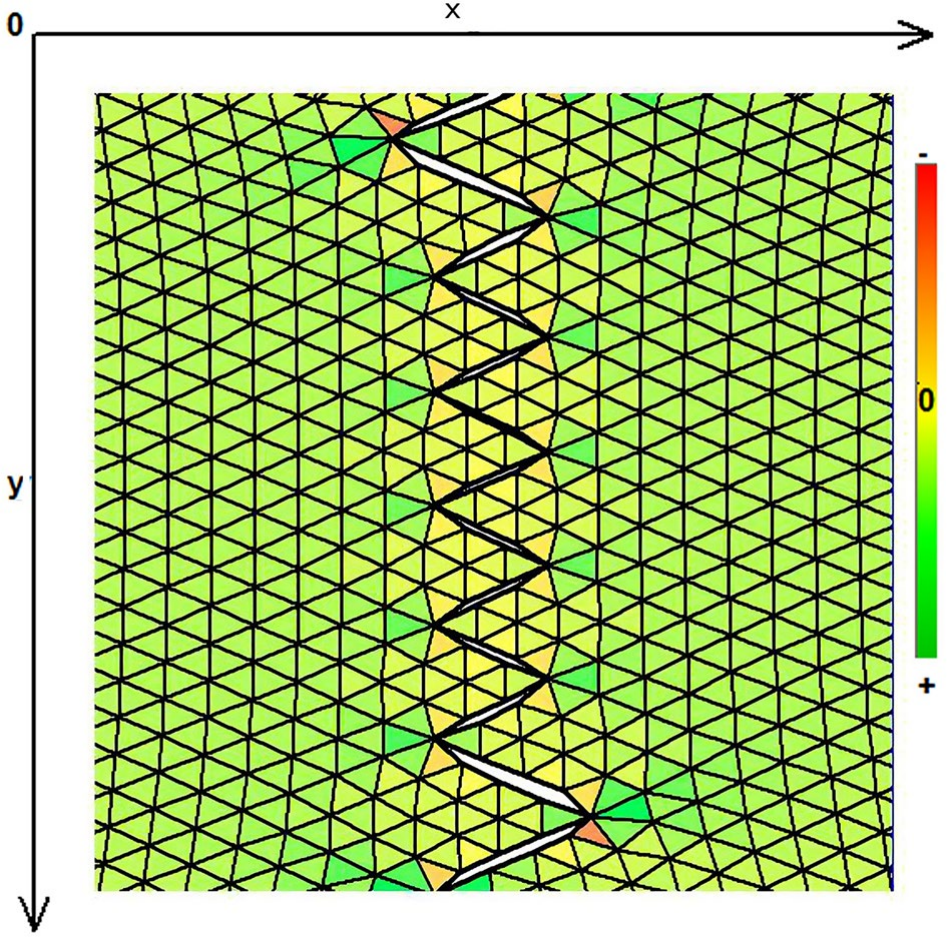
The FE model: stress along the x-axis, zoom at the center after closure.

**Figure 10:**
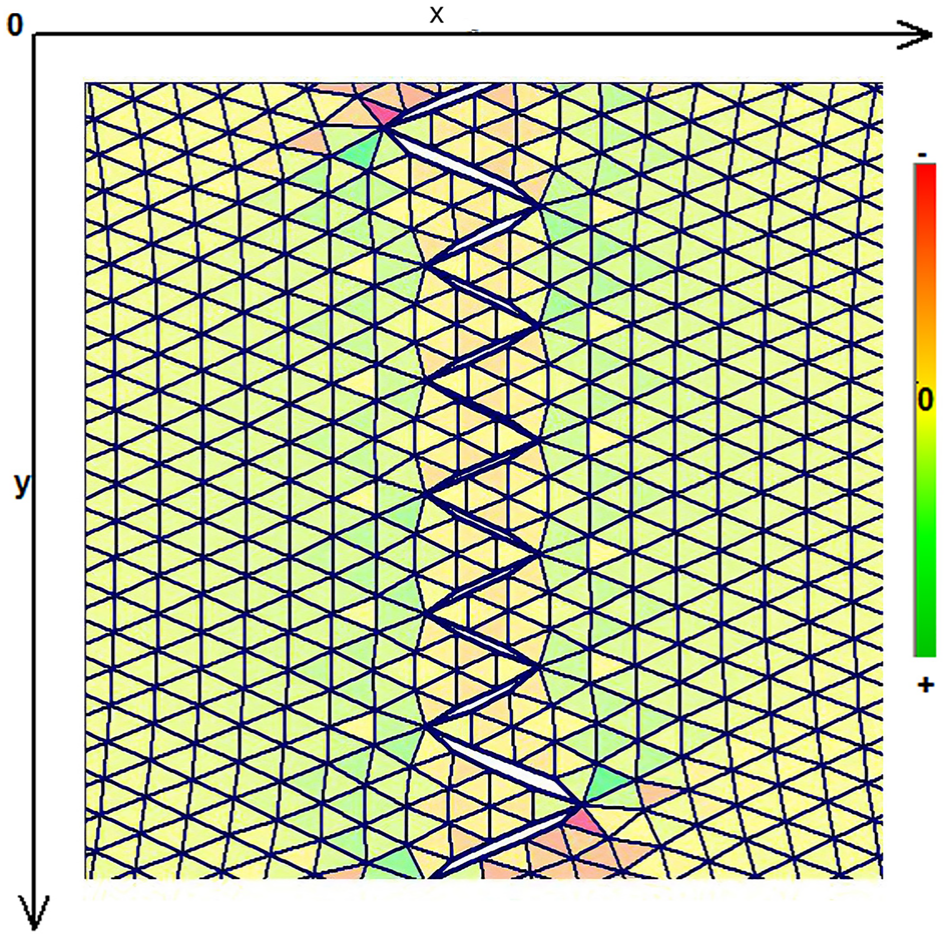
The FE model: stress along the y-axis, zoom at the center after closure.

**Figure 11:**
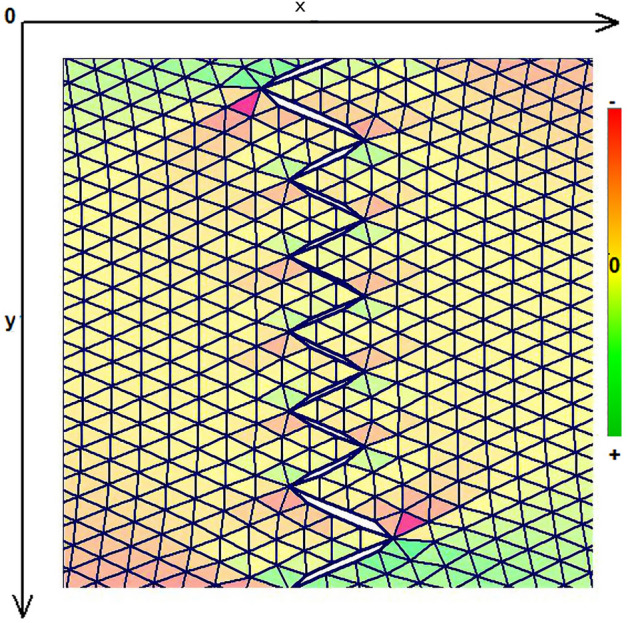
The FE model: shearing Txy stress, zoom at the center after closure.

**Figure 12:**
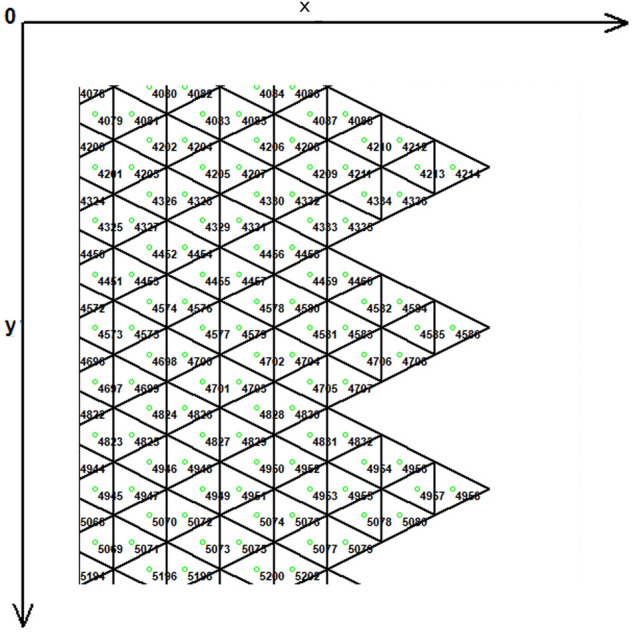
Central elements.

**Figure 13:**
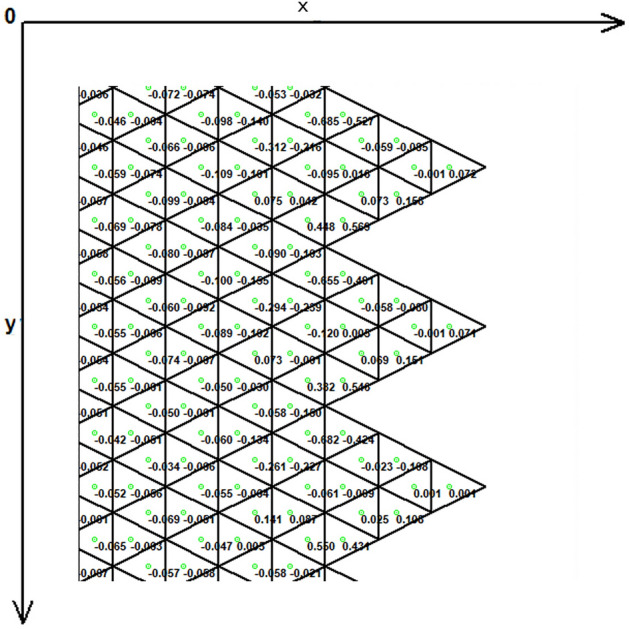
Shear τxy stress.

**Figure 14:**
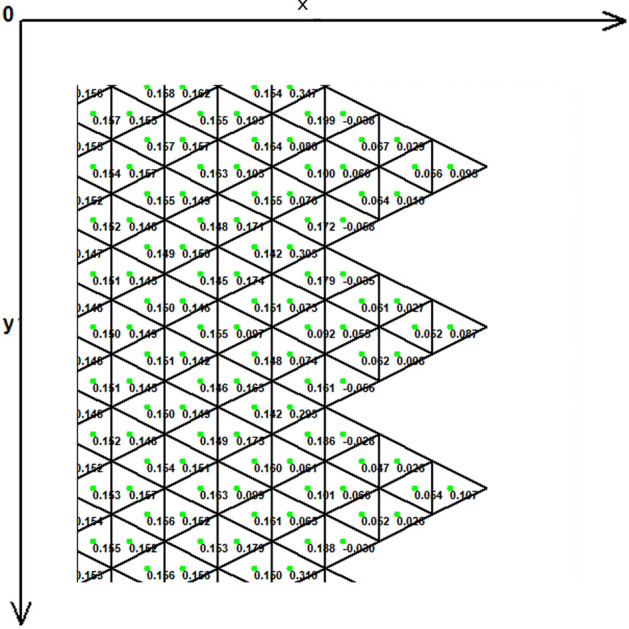
Strain along the x-axis.

**Figure 15:**
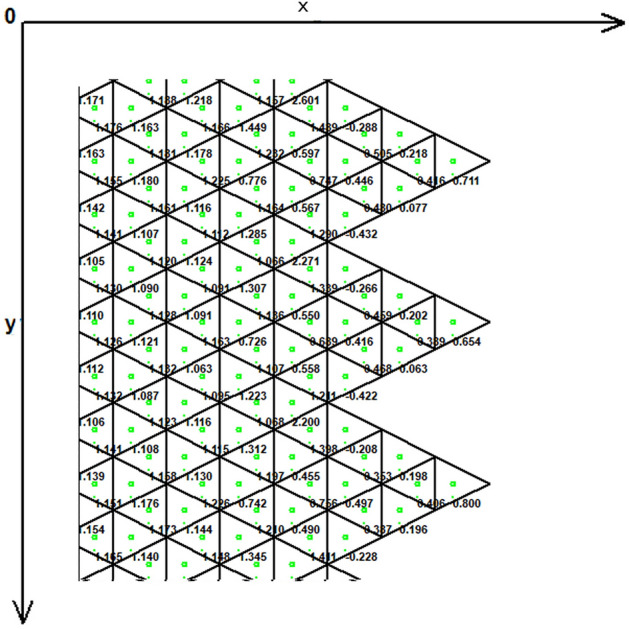
Stress along the x-axis.

**Figure 16:**
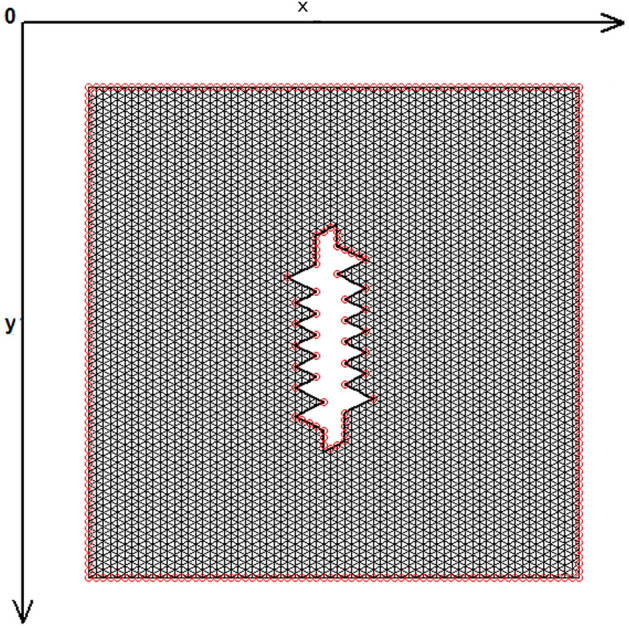
Boundary conditions of the model.

As expected, the maximum compressive stresses developed at the edges of the wound as shown in Figs. 4, 5 and 6. Figures 4 and 5 demonstrate the stress along the X-axis and the Y-axis, respectively. Figure 6 shows the shear stress along the xy axis. Compressive stresses are depicted in red, normal stresses are depicted in green and the yellow areas represent low to zero stress areas. Increased stresses (green), particularly along the X-axis where the stitching was performed, developed only near the two ends of the stitching line and behind the triangular flaps of the W-plasty. Interestingly, in the triangular flaps, the stresses were clearly lower than those of their neighboring areas. The maximum compressive stresses in the 2D model we used, correspond to the dog ears in the 3D model. Figures 7, 8, 9, 10 and 11 demonstrate the stress per segmented area. Figure 12 shows the central elements and Fig. 13 shows the distribution of the τxy stress in the central elements. Figures 14 and 15 demonstrate the strain and the stress along the x-axis, respectively. Figure 16 shows the fixed nodes in the periphery of model, while the others in the centre are moving to close the deficit.

**Figure 17:**
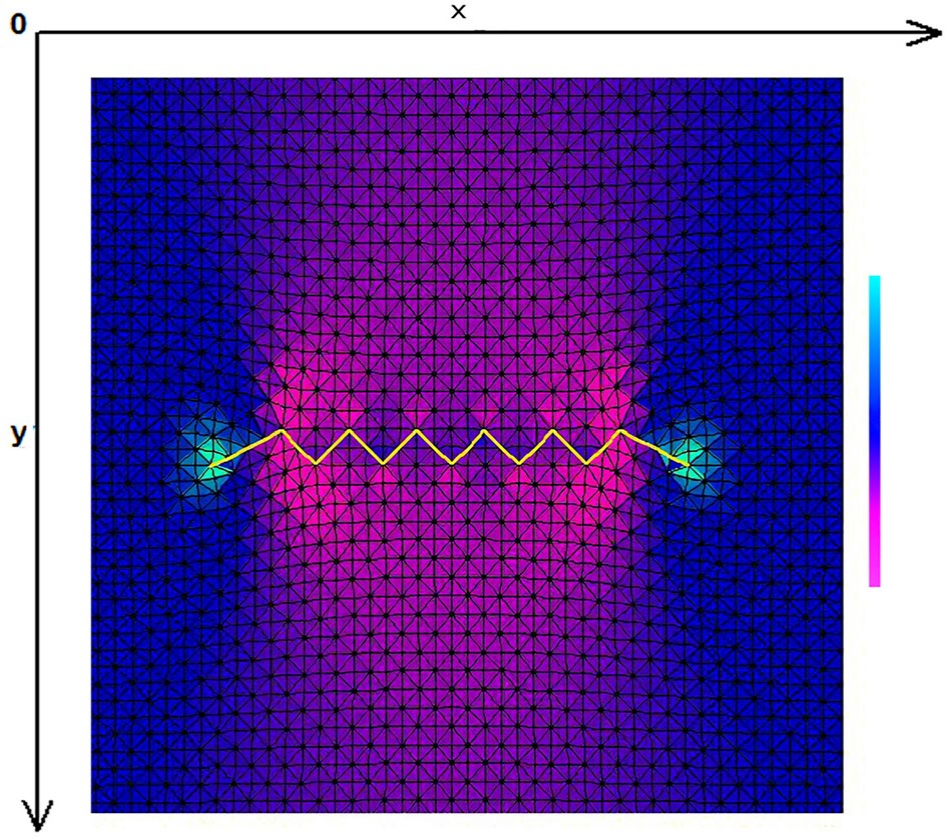
Another FE model with following characteristics: W-plasty angle 90 degrees, 2332 nodes, 4416 elements and 8 elements per flap, with the stitching being done along the y-axis. Compressive stresses are illustrated in green, normal stresses in red, and low to zero values in dark blue.

Indeed, the X and Y axis stresses and shearing stresses Txy in the elements involved in the broken stitching line, show lower stresses than the elements behind the stitching line. We confirmed this finding in several finite element models with different number of elements and different W-plasty angles. In all cases we had the same results. Figure 17 demonstrates another model with following characteristics: W-plasty angle 90 degrees, 2332 nodes, 4416 elements and 8 elements per flap, with the stitching being done along the y-axis. Compressive stresses are illustrated in green, normal stresses in red, and low to zero values in dark blue. The tension stress is distributed along the y axis and not in the triangular flaps, which demonstrate low to zero stresses.

## Discussion

Despite the improvement of scars with time and the huge variety of pharmaceutical means, scars remain a main source of discomfort for the operated patient, which prompts him/her to visit a plastic surgeon. W-plasty has been applied since decades in facial plastic surgery and remains the first-line treatment modality for repairing malformed facial scars. Its ‘broken-line’ or ‘accordion’ appearance allows the scar to break down into smaller triangles, with at least one side parallel to a poorly defined relaxed skin-tension line (RSTL), which causes light scattering and leads to less conspicuous scars^5^.

It is well known that the skin is a highly anisotropic material^16,17^. If a wound crosses the dynamic lines of the skin, the resulting scar may not be the best. Borges, as well as subsequent researchers, reported poor wound orientation in relation to the dynamic lines and Langer lines to be the main cause of bad scars. The explanation is that these scars developed perpendicular to the dynamic lines of the skin, and, therefore, created high tensions, which resulted in poor outcomes. Anisotropy refers to the different elastic properties along the different axes of the skin, i.e. when stretching the tissue in parallel direction, the fibers become straight at lower stretch levels^16^. The degree of anisotropy of the mechanical properties with respect to the tension lines has been quantified^17^.

Today, the finite element method models complex procedures and techniques in three dimensions so that it is a powerful teaching tool in the plastic surgery of skin flaps^18,19^. However, in addition to the application to skin flaps, finite element analysis is also applied to the skeleton of the craniofacial complex^20^. FE models, despite their limitations, describe the influence of individual surgical variables on tissue biomechanics and assist flap understanding^15^.

We, herein, presented an explanation for the effectiveness of the W-plasty method based on a finite element model. According to our findings, the good outcomes of the W-plasty should be attributed to the method itself, which distributes the tension stress to the elements behind rather than to the elements of the triangular flaps. These findings should be viewed in light of the material definition limitations.

In our model, we found reduced stresses in the triangular flaps in several models and variations. There is no consensus regarding the optimal W-plasty design. Borges recommends the triangular flaps being in the form of isosceles triangles each with approximately 6 ram. at its base, 6½ mm. in height, two 65 degree angles and one 50-degree angle. Sharper flaps require more tissue to be excised, are more difficult to suture and may impair circulation, while less sharp flaps result to scars that follow less the tension lines^2^. According to Jáuregui et al., a 1.2- to 1.5-cm pedicle width may easily be closed primarily. It is recommended that the peaks and troughs fall as much as possible within the natural rhytids and, if possible, that the individual limbs approximate 5 mm^6^. Goutos et al. propose two main designs of either the isosceles/equilateral or the scalene triangle W-plasty. The former is suitable for regions with curved surfaces lacking clear RSTLs, while the latter is recommended for regions with well defined RTSL, e.g. nasolabial region and forehead^8^.

Z-plasty surgery and W-plasty surgery are used alternatively in the restoration of scars. Where Z-plasty surgery is applied it does not create a defect unlike W-plasty which creates a defect and causes an increase in tension. Z-plasty is mainly preferred in ricnotic scars and is applied to resolve them by redirecting the triangular flaps, especially when they intersect with joints. There are also cases where both techniques can be used together. Wave resections or irregular line resections have also been proposed to treat scars. Such resections are based on the same principle as W-plasty, i.e. increasing the incision length to create more load points. W-plasty has the advantage of being more predictable than that of using wavy lines of closure to the point that specific plastic patterns have been devised to design W-plasty in the skin.

While our study sheds light on the multifaceted effectiveness of W-plasty in wound closure, it is crucial to acknowledge the need for future investigations to delve deeper into the dynamic interplay of various factors. For instance, considering the complexities of real-world surgical scenarios, incorporating factors like patient-specific anatomical variations, tissue properties, and wound characteristics into the finite element model could provide a more comprehensive understanding. Furthermore, the integration of advanced imaging techniques, such as MRI or CT scans, could offer more accurate representations of real tissue behavior during wound closure. This could potentially enhance the precision and fidelity of our finite element simulations. Additionally, extending the study to encompass a broader range of wound sizes and shapes would provide a more nuanced understanding of the technique’s applicability across various clinical scenarios. Moreover, investigating the impact of suturing techniques and materials in conjunction with W-plasty could unveil synergistic approaches for optimized wound closure outcomes.

Our study has several limitations: (a) *Simplified Model Assumptions:* The finite element method relies on certain assumptions and simplifications to model complex biological processes. In this study, the model assumes perfect elasticity of the membrane and may not fully capture the intricacies of real biological tissues, (b) *Uniform Material Properties:* The model assumes uniform material properties for the elastic membrane, which may not accurately represent the heterogeneous nature of actual biological tissues, (c) *Neglect of Biological Variability:* The study does not account for individual patient variability in skin elasticity, thickness, or other anatomical factors that could affect wound closure outcomes, (d) *Stitching Technique Standardization:* The study assumes a uniform stitching technique across simulations. In reality, surgical techniques can vary widely, potentially influencing the effectiveness of the w-plasty closurem, (e) *Static Model:* The model does not consider dynamic factors such as tissue movement, muscle contractions, or changes in tension over time, which are inherent in real-life wound healing scenarios, (f) *Idealized Geometries:* The model uses simplified geometries that may not fully represent the irregular shapes and contours of actual wounds, (g) *Exclusion of Clinical Variables:* Clinical factors such as patient age, comorbidities, and wound location, which can influence wound closure outcomes, are not considered in the model and (h) *In Vitro versus In Vivo:* The findings of this study are limited to in vitro simulations and may not directly translate to in vivo wound closure scenarios due to the controlled laboratory conditions.

We conclude that the effectiveness of W-plasty, as shown using the finite element method, should be attributed not only to the scar orientation in relation to the relaxed tension skin lines but also to the special design of the triangular flaps used. This finding assists the general understanding of the method and should be taken into account by the clinician during flap designing.

## Data Availability

The datasets used and/or analysed during the current study available from the corresponding author on reasonable request.
